# Global genomic epidemiology of blaGES-5 carbapenemase-associated integrons

**DOI:** 10.1099/mgen.0.001312

**Published:** 2024-12-04

**Authors:** William Matlock, Liam P. Shaw, Nicole Stoesser

**Affiliations:** 1 Nuffield Department of Medicine, University of Oxford, Oxford, UK; 2 Department of Biology, University of Oxford, Oxford, UK; 3 NIHR Health Protection Research Unit in Healthcare Associated Infections and Antimicrobial Resistance, University of Oxford, Oxford, UK; 4 Oxford University Hospitals NHS Foundation Trust, Oxford, UK

**Keywords:** beta-lactamase, GES, mobile genetic elements, *Pseudomonas aeruginosa*

## Abstract

Antimicrobial resistance (AMR) gene cassettes comprise an AMR gene flanked by short recombination sites (*attI* and *attC* or *attC* and *attC*). Integrons are genetic elements able to capture, excise and shuffle these cassettes, providing ‘adaptation on demand’, and can be found on both chromosomes and plasmids. Understanding the patterns of integron diversity may help to understand the epidemiology of AMR genes. As a case study, we examined the clinical resistance gene *bla*_GES-5_, an integron-associated class A carbapenemase first reported in Greece in 2004 and since observed worldwide, which to our knowledge has not been the subject of a previous global analysis. Using a dataset comprising all de-duplicated NCBI contigs containing *bla*_GES-5_ (*n*=104), we developed a pangenome graph-based workflow to characterize and cluster the diversity of *bla*_GES-5_-associated integrons. We demonstrate that *bla*_GES-5_-associated integrons on plasmids are different to those on chromosomes. Chromosomal integrons were almost all identified in *Pseudomonas aeruginosa* ST235, with a consistent gene cassette content and order. We observed instances where insertion sequence IS*110* disrupted *attC* sites, which might immobilize the gene cassettes and explain the conserved integron structure despite the presence of *intI1* integrase promoters, which would typically facilitate capture or excision and rearrangement. The plasmid-associated integrons were more diverse in their gene cassette content and order, which could be an indication of greater integrase activity and ‘shuffling’ of integrons on plasmids.

**Impact Statement**

There is a great deal of interest in the genomic epidemiology of AMR genes. However, it has become increasingly clear that traditional reference sequence-based approaches are inadequate due to the plastic nature of mobile genetic elements associated with AMR gene spread. Whilst substantial efforts have been made to improve plasmid genomic epidemiology, integrons have been neglected, along with the AMR genes associated with them. Here, we explore the potential for pangenome graphs to improve the genomic surveillance of integron-associated AMR genes. Our study focuses on *bla*_GES-5_, a carbapenemase first reported in Greece in 2004 and since observed worldwide. Despite its widespread presence, *bla*_GES-5_ has not been the subject of a comprehensive global genomic analysis until now.

## Data Summary

Analysis scripts and all 431 contigs from the National Centre for Biotechnology Information (NCBI) database are available at https://github.com/wtmatlock/ges. NCBI accessions for deduplicated contigs are given in Table S1, available in the online version of this article.

## Introduction

The dissemination of antimicrobial resistance (AMR) genes combines multiple modes of transfer, operating within a hierarchy of genetic units: AMR genes, their immediate genetic contexts, mobile genetic elements (MGEs), the bacterial host and microbial communities [1], similar to the structure of a nested ‘Russian doll’ [2]. The combined actions of these units mean that the epidemiological picture of AMR gene dissemination can quickly become complex.

**Fig. 1.**
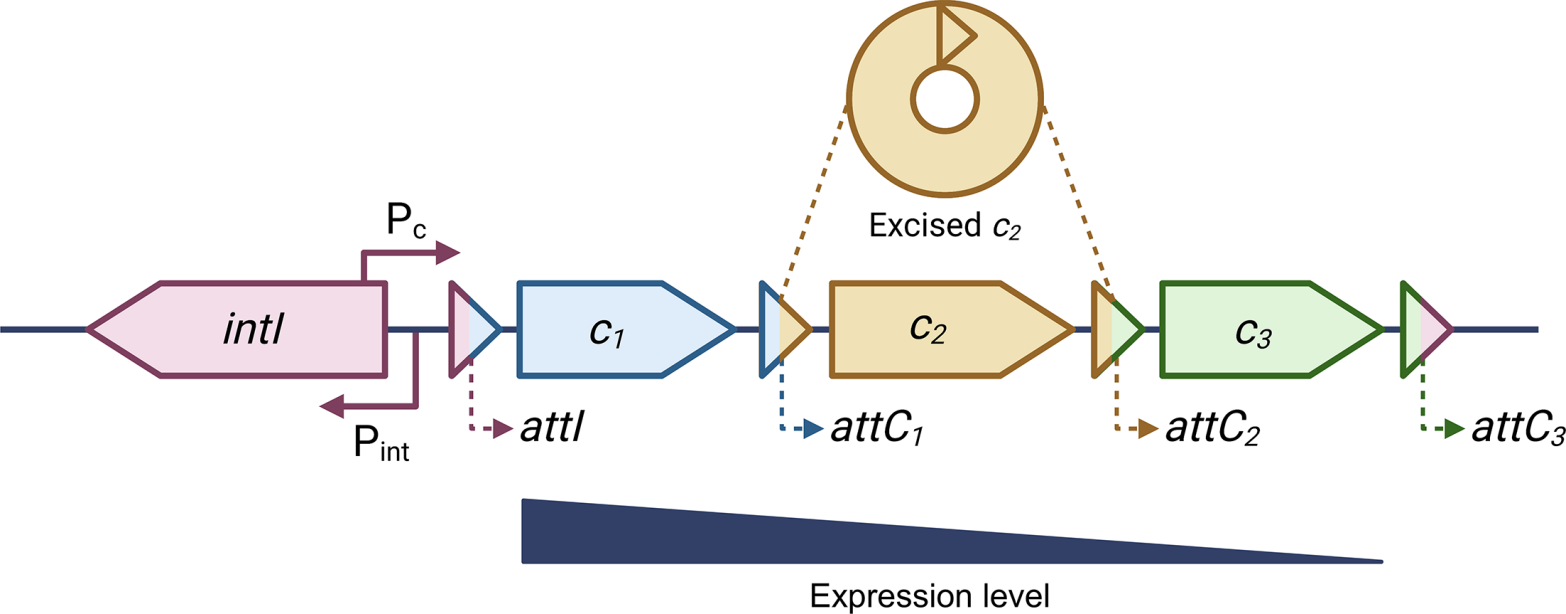
A schematic of an integron. Adapted from Gillings 2017 [75] and Ghaly 2017 [11]. Shown is an integrase in the reverse orientation with a P_c_ and P_int_. This can lead to transcriptional interference [8]. Also shown are three gene cassettes (*c_1_*, *c_2_* and *c_3_*), flanked by the attachment sites (*attI*, *attC_1_*, *attC_2_* and *attC_3_*). Gene cassette *c*_*2*_ has been excised above the integron. The expression of the gene cassettes generally decreases with distance from the promoter P_c_.

The immediate genetic context, or ‘flanking sequences’, of AMR genes can act as epidemiologically relevant markers. These sequences can contain a great deal of genetic diversity, encompassing an array of other genes, MGEs and other structures, including integrons [3]. Structurally, class 1 integrons consist of an integrase (*intI1*) upstream of an array of gene cassettes. Each gene cassette is formed of one of more cargo genes, and flanked by recombination sites (*attI* and *attC* or *attC* and *attC* sites in the first and subsequent positions, respectively; Fig. 1). The integron integrase belongs to the family of site-specific tyrosine recombinases, including phage integrases such as Frat1 and D29 and XerC and XerD in *Escherichia coli* [4]. Whilst most genes in this family share the conserved residue RHRY, alongside other motifs (boxes I and II and patches I, II and III), integron integrases uniquely possess an additional ~30 residues near the patch III motif [5], which is thought to aid the movement of gene cassettes [6]. This behaviour also enables the integron to vary the order of the gene cassettes, which in turn modulates their relative expression by proximity to the cassette promoter (P_c_; found within the integrase) [7]. This variable order permits ‘adaptation on demand’ in response to changing conditions, with the array of cassettes acting as a store of previously beneficial genes [8]. The expression of the integrase is controlled separately by its integrase promoter (P_int_; within or downstream of the integrase). Class 1 integrons are found on both chromosomes and plasmids (known as ‘mobile integrons’) [3]. On plasmids, the integron can be transferred between cells. Then, within the new cell, the gene cassettes can be captured by other integrons [9]. In this way, plasmids and integrons work in tandem to disseminate AMR genes.

Class 1 integrons are associated with AMR genes conferring resistance to several classes of antibiotics, including aminoglycosides (AAC/AAD-family-resistant *N*-acetyltransferases), beta-lactams (GES/IMP/VIM-family beta-lactamases), trimethoprims (Dfr-family dihydrofolate reductases) and sulphonamides (Sul-family dihydropteroate synthases) [3]. Class 1 integrons are believed to have jumped from environmental to human species via a Tn*402* transposon [10,11]. When sulphonamides were widely used antibiotics (discovered in the mid-1930s), it gave any environmental integrons with a *sul1* gene a selective advantage in clinical contexts. Alongside *sul1*, early clinical class 1 integrons also carried the *qacE* gene (conferring resistance to biocides), and a mercury resistance *mer* Tn*501*-like transposon [11]. The structures of contemporary class 1 integrons are still associated with many of these structures downstream of the gene cassettes in a 3′ conserved sequence [12], as well as the whole integron being flanked by the inverted repeats associated with Tn*402* [13]. However, class 1 integrons also carry a diversity of more recently acquired gene cassettes [14]. Currently, integron integrases are so prolific in human microbiota that they have been used as a proxy for anthropogenic pollution [15]. Class 2, 3, 4 and 5 integrons are also observed in both clinical and environmental niches and are associated with different integrases and gene cassette arrays [9,12, 14, 16].

Currently, there are several software tools for the epidemiological study of AMR. These include those focussing on non-plasmid MGEs such as transposable elements [17,18], as well as whole plasmids [19,21] and bacterial strains that commonly carry particular AMR genes [22]. For integrons, epidemiology has often relied on sequence comparisons to reference databases, such as INTEGRALL [14], so is restricted to known diversity. Moreover, due to the rearrangement of gene cassettes, integrons containing the same cassette array but in a different order can have poor pairwise alignment scores (for example, with MAFFT [23]), diminishing the utility of traditional alignment-based methods. Lastly, the repetitive structures of integrons make sequence assembly challenging, often generating short or ‘broken’ contigs [24,26].

Alternatively, integron epidemiology can use phylogenetic-based approaches. Integron integrases are highly conserved genes [5,27], with a strong phylogenetic signal for host genera and sampling environment [28,30]. This signal has similarly been demonstrated for *attC* site variants and is arguably connected: both the integrase and *attC* sites must cooperate well to successfully modulate the gene cassette array under selective pressure [31]. Previous studies have demonstrated the potential of tracking single-nucleotide variants (SNVs) of the whole integron sequence across a single outbreak [32] and synonymous single-nucleotide variants (sSNVs) of individual gene cassettes across GenBank sequences [3]. Although synonymous mutations might leave the protein unchanged, they can still impact fitness [33], for instance, by increasing translational efficiency [34,35]. Within a bacterial population, tracking SNVs might indicate whether an integron has emerged locally or has been imported, hence improving our understanding of how specific integrases and gene cassettes have moved historically. This is particularly important for mobile integrons. Overall, reference-free methods that are robust to both variable gene cassette synteny and ‘broken’ contigs would be a useful addition to studying integron and AMR gene epidemiology.

In this study, we describe a novel computational workflow for integron epidemiology. Capturing the diversity and synteny of gene cassettes in a sample of genomes has parallels to pangenome graph construction, where the aim is to summarize the sharing of genetic structures within whole genomes. Here, we leverage recent advances in pangenome graphs to cluster a large dataset of sequences containing a specific integron-associated AMR gene, *bla*_GES-5_. We show that this approach is concordant with genetic structure as well as bacterial niche and that it is robust to both variable gene cassette synteny and the inclusion of short or incomplete contigs. In addition, we examine the potential of SNV profiling for global integron epidemiology.

## Methods

The GitHub repository https://github.com/wtmatlock/ges contains a tutorial for the scripts and commands used in this analysis.

### Distribution of NCBI GES variant annotations

All GES genes (*n*=57) were retrieved from the NCBI Reference Gene Catalogue (as of 17 February 2023). All alleles were 864 bp except GES-42 (NG_065870.1 : 1-873) with a 9 bp insertion. Protein sequences were aligned using ClustalW [36] (v 2.1; ‘slow’ mode), which was also used to produce a neighbour-joining tree, correcting for multiple substitutions. The hydrolytic profile was determined from experimental studies, collated by the beta-lactamase database (BLDB) [37] (as of 17 February 2023). NCBI GES variant genera distribution was determined by querying the NCBI’s ‘Pathogen Detection Microbial Browser for Identification of Genetic and Genomic Elements’ (MicroBIGG-E) service [38] using the query ‘element_symbol:blaGES*’ (as of 27 February 2023).

### Dataset curation

Using the same query as above, 1375 contigs were retrieved from the NCBI’s MicroBIGG-E service (as of 27 February 2023). These contigs were originally annotated using AMRFinderPlus [39], a BLAST-/HMMER-based program reliant on the NCBI Pathogen Detection Reference Gene Catalogue. Contigs were then annotated for *bla*_GES-5_ using the NCBI GES-5 protein reference sequence WP_012658785.1 and BLASTX [40]. Filtering by 100% amino acid identity/coverage and a single hit yielded 431 contigs.

NCBI BioProjects can contain multiple isolates from clonal strains and outbreaks, as well as from more general repeat sequencing. This meant that there were potential biases in 431 *bla*_GES-5_-positive contigs, which could lead to the artificial inflation of some flanking structures and/or isolate metadata in the downstream analysis. To help correct this, the contigs were deduplicated as follows (see deduplication.R): first, the longest contig from each BioSample was kept, and the rest discarded. If there were ties for the longest contig, a random representative was chosen, yet this was never the case. Then, within each BioProject, contigs that were perfectly contained in another were discarded to diminish the bias of flanking sequence duplicates with the same metadata. Pairwise contig containment was calculated using Mash screen [41], with code given in the repository. If this resulted in all contigs from a BioProject being discarded, we chose a random, longest representative. This left 107 contigs. Finally, 3 contigs were removed from the dataset because they contained repeated *bla*_GES-5_ PanGraph blocks in the 10 kbp flanking sequences: adjacent blocks in NZ_CP073313.1 and NZ_VAAM01000031.1 and ~9 kbp apart in NZ_UARQ01000013.1, with several other PanGraph blocks repeated. Whilst these cases were potentially assembly errors, they also made pangenome graph construction challenging. This left a total of 104 contigs for analysis.

Contig lengths were calculated with fastaLengths.py in the repository, which depends on Biopython [42]. BioProject accessions were retrieved using the NCBI’s E-utilities [43] via contig BioSample accessions. The code used is given in the repository README.md tutorial. Pairwise sequence containment used Mash screen [41] (v. 2.2, default parameters except sketch size -s 100000).

### Exploring *bla*_GES-5_ flanking sequences with Flanker

Flanking sequences were extracted and clustered using the Python tool Flanker with a custom Abricate (v. 1.0.1; Seeman 2020) database containing only the NCBI *bla*_GES-5_ nucleotide reference sequence NG_049137.1. The Flanker command used was flanker --gene GES-5 --database GES-5 --fasta_file ./“$f”.fasta --flank both --window 10000 --wstop 10000 --wstep 100 --include_gene --cl. Flanker was run by the Slurm scheduler script runFlanker.sh but could be adapted to any command line.

The Flanker output was then processed (see plotFlanker.sh). First, matrices were generated for both the upstream and downstream flanks, where each row represented a contig and each column the flanking sequence length (100 bp, 200 bp, …, 10 000 bp). Each entry was either (i) a unique Flanker cluster label or (ii) 9999 if the flanking sequence had no cluster label because it was too short. Then, the row-wise Jaccard index was calculated to generate a distance matrix, which was hierarchically clustered. This generated the ‘meta-clusters’ (a clustering of the Flanker cluster profiles); 20/104 and 7/104 sequences were removed upstream and downstream, respectively, because they had insufficient flanking sequences to be clustered.

### Sequence annotations

Contig annotations were pre-generated by the NCBI’s Prokaryotic Genome Annotation Pipeline [44] and retrieved via contig accessions. The code used to download the GFF files is given in the repository README.md tutorial. Additional annotations of integron integrases, *attC*/*attI* sites and integron promoters P_c_/P_int_ were generated using IntegronFinder [45] (v. 2.0.2; default parameters except --local-max --promoter-attI --lin; see runIntegronFinder.sh). Integron finder annotates integrases using hidden Markov model protein profiles and *attC* sites using covariance models to predict secondary structure. For *attI* sites and promoters, reference motifs are searched for in query sequences. Integrase type (*intI1* or *intI3*) was confirmed using BLASTX [40]. Lastly, transposase annotations were generated with Abricate [46] (v. 1.0.1) with the ISfinder database [47] (as of 28 February 2023) using default parameters except --db isfinder (see runISfinder.sh).

### Clustering *bla*_GES-5_ flanking sequences with PanGraph

The 10 kbp *bla*_GES-5_ flanking sequences (extracted with Flanker as described above) were passed into PanGraph [48] (v. 0.6.3) with pangraph build and then pangraph export --edge-minimum-length. BLASTN [49] (v. 2.5.0) determined which of the PanGraph blocks contained NG_049137 (see runPanGraph.sh). An additional post-processing script converted the PanGraph GFA output to a visualization of coloured linear blocks (see pangraphGFA.py).

### Determining contig origin

Contigs were classified as chromosomal if they could be typed with mlst [50] (v. 2.23.0) using default parameters (see runMlst.sh). Contigs were classified as plasmid if they could be typed with either Abricate [46] (v. 1.0.1) and the PlasmidFinder database [19] (as of 28 February 2023) using default parameters except --db plasmidfinder (see runPlasmidFinder.sh) or MOB-Typer [51] (v. 3.1.4) using default parameters (see runMobTyper.sh). Contigs were left unclassified if none of these methods gave a typing.

### SNV profiling

First, the integrase annotation positions were extracted from the IntegronFinder output. Then, the integrase sequences were retrieved from the full contigs using extractIntegrase.py in the repository. Integrase sequences were aligned using ClustalW [36] (v. 2.1) in slow/accurate mode. The SNP distance matrix was generated using snp-dists [52] (v. 0.8.2) with default parameters, and SNP constant sites were found using SNP-sites [53] (v. 2.5.1) with -C and otherwise default parameters. The SNP clustering/heatmap was generated using the script plotHeatmap.R. The method was identical for *bla*_GES-5_, except the sequences were instead extracted using runFlanker.sh but altering the window to 0 bp with --window 0.

### Data visualization

Figs 2, S1 and S3–5 were generated using ggplot2 [54] in R. Fig. 1 was drawn using BioRender.com. Fig. S2 was produced using Bandage [55]. Fig. 3 was plotted using plotFlanker.R. For plotting Fig. 4, as well as statistics for contigs/annotations/PanGraph, we used plotFlanks.R.

**Fig. 2.**
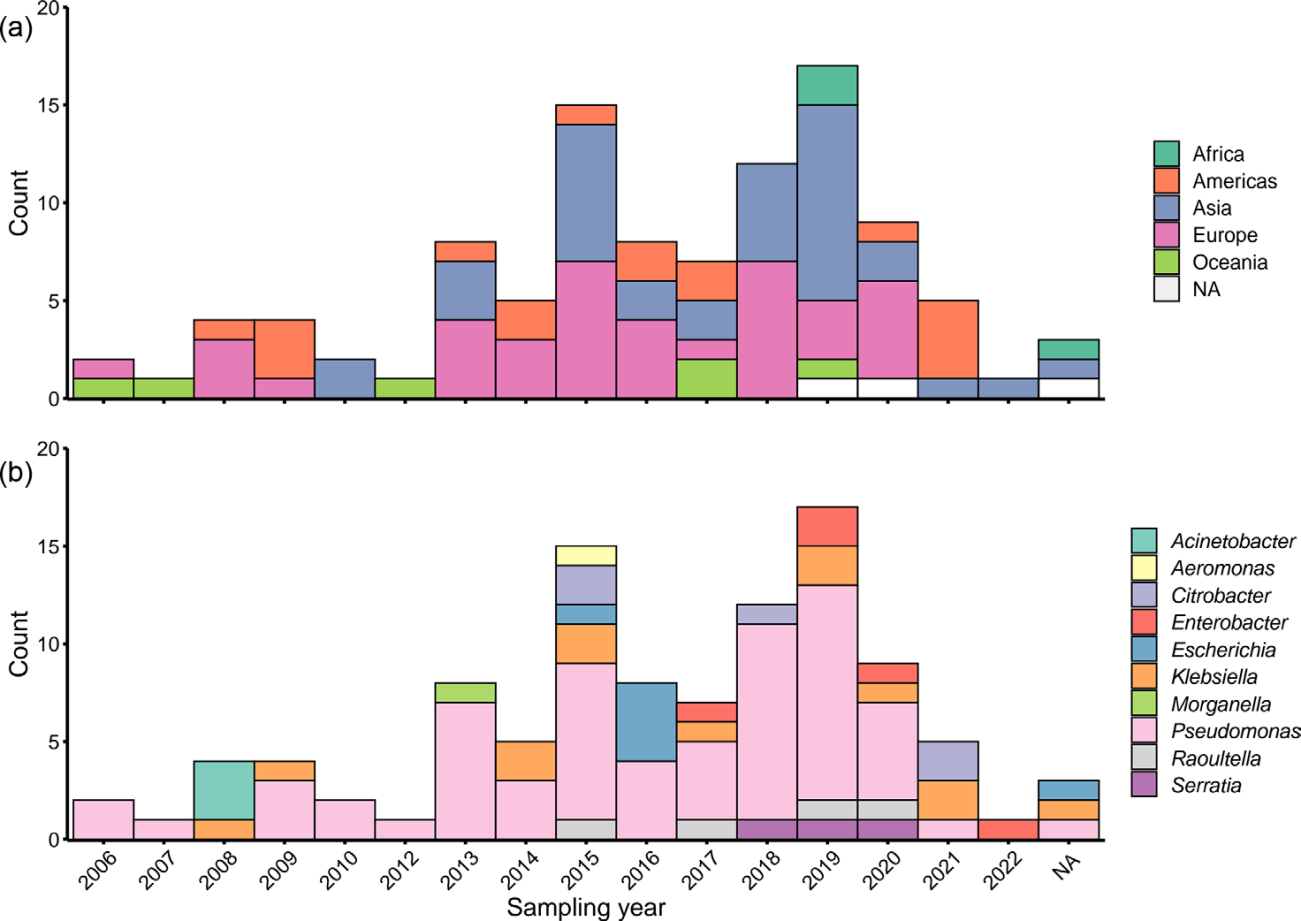
NCBI metadata distributions for the 104 bla_GES-5_-positive contigs. (**a**) Shows ISO region and (**b**) isolate genus by sampling year. Note, no contigs were sampled in 2011, so this year is omitted from the *x*-axis.

**Fig. 3.**
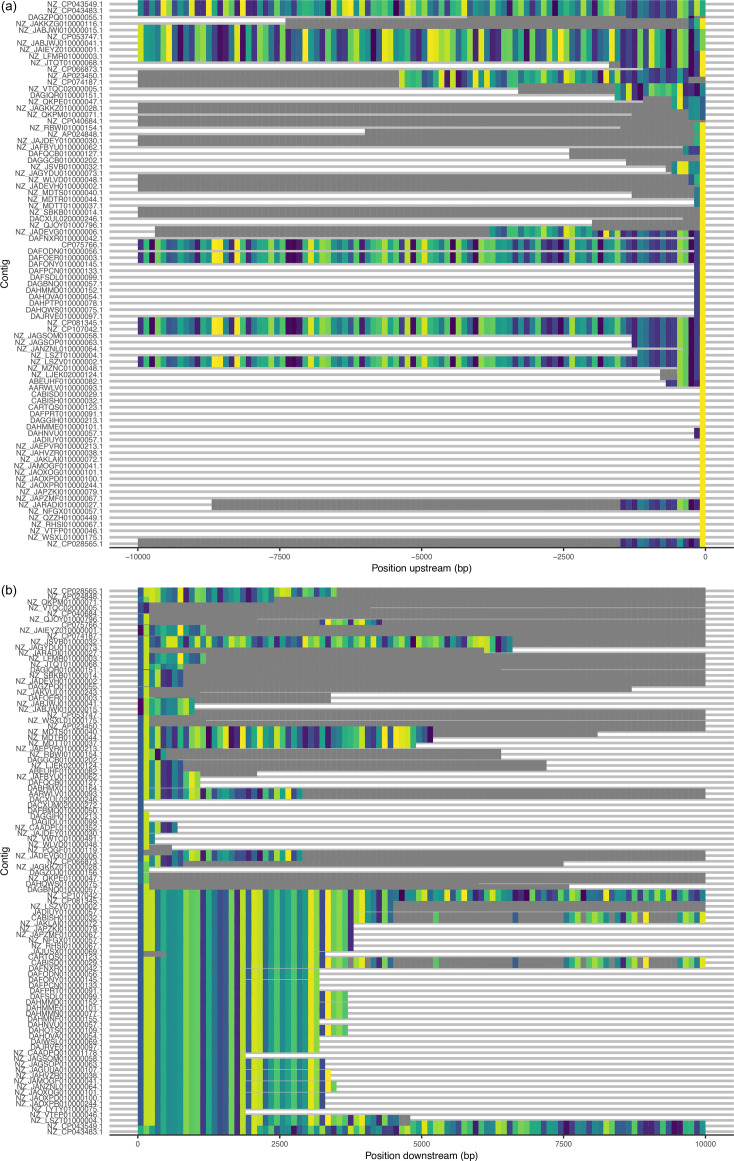
Exploration of flanking sequence diversity. (**a**) Upstream Flanker output, from 100 bp flanking sequences to 10 000 bp flanking sequences in 100 bp windows. For each length, tile colours represent clusters found multiple times, and grey values represent unique clusters. Colours do not compare across lengths, only within lengths. (**b**) Downstream Flanker output, as described for the upstream flanking sequences.

**Fig. 4.**
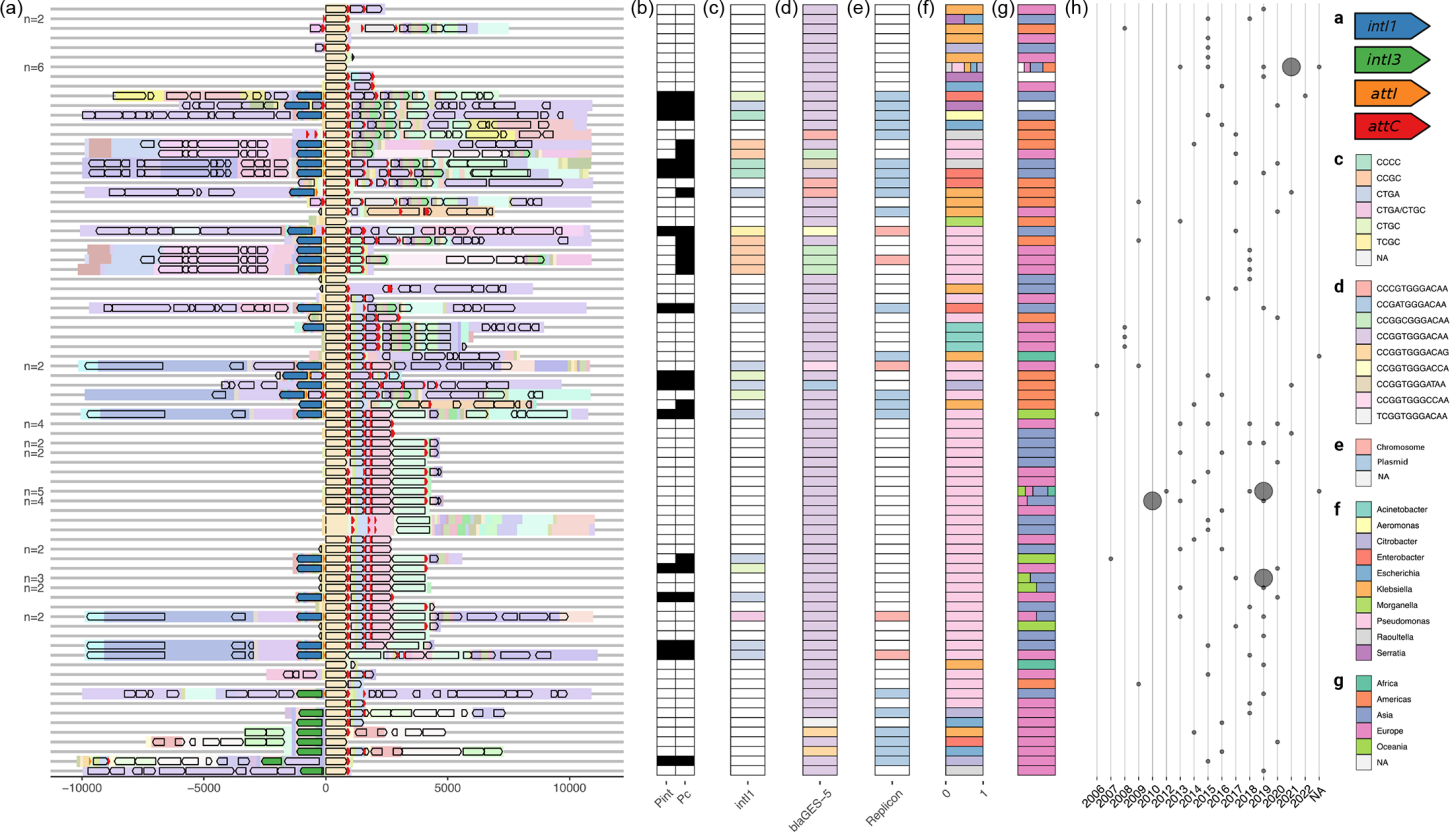
10 000 bp flanking sequence analysis for *bla*_GES-5_. (**a**) Clustering-ordered PanGraph blocks (pastel colours) overlaid with CDS annotations (black outlined arrows) and integron-specific annotations: *intI1* in blue, *intI3* in green, *attI* in orange and *attC* in red. Numbers to the left indicate duplicates, with boundaries indicating where the coloured PanGraph blocks begin/end. (**b**) P_int_ and P_c_ presence (black) or absence (white). (**c**) *intI1* SNV profiles, corresponding to the blue *intI1* annotations in panel (**a**). (**d**) *bla*_GES-5_ SNV profiles. (**e**) Contig origin (chromosomal, plasmid or na). (**f**) Contig genus distribution. (**g**) Contig ISO region distribution. (**h**) Contig sampling year distribution. Smaller circles indicate one isolate, and larger circles indicate two isolates.

## Results

### A historical and global dataset of *bla*_GES-5_ contigs

Public sequence databases such as the NCBI present a valuable resource for the study of AMR gene epidemiology, despite potential sampling biases. The historic and continued deposition of sequence data means that exploratory studies can represent a global epidemiological picture. For example, a recent study leveraged the NCBI to analyse over 7000 *bla*_NDM_-positive contigs, demonstrating the joint role of Tn*125*, Tn*3000* and IS*26* in the gene’s global spread [56].

The GES family of class A beta-lactamases contains 57 protein variants as of July 2023 [37]; the first of which, GES-1, was reported in 1999 on a 140 kb plasmid in a *Klebsiella pneumoniae* [57]. GES variants are usually found as class 1/3 integron gene cassettes [58], and many demonstrate carbapenemase activity [37]. This analysis focuses on *bla*_GES-5_ since it is both a carbapenemase and the most abundant GES enzyme annotated in NCBI sequence data (Fig. S1 in Supplementary Material; see Methods). To date, *bla*_GES-5_ has been linked to several clinical outbreaks in Japan [59], Korea [60], the UK [61] and the Czech Republic [62]. Moreover, *bla*_GES-5_ has been identified globally across a range of species, both on the chromosome (most notably *Pseudomonas aeruginosa* ST235 [63]) and plasmids (for example, the Q-type plasmid pHPRU111 found across several *Enterobacterales* spp. [61]). It is also possible that the abundance of GES-5 and other GES variants is underestimated due to a lack of targeted assays [64,65].

We first downloaded all 1375 contigs annotated by the NCBI with a GES variant (see Fig. S1). We then annotated these for *bla*_GES-5_, giving 431 contigs. Finally, we then deduplicated to 107 to control for outbreaks/isolate repeat sequencing (see Methods). We also removed three contigs that we found complicated the subsequent pangenome graph construction (see Methods), leaving a total of 104 contigs (see Table S1). Our deduplicated dataset spanned 2006–2022 (*n*=3 missing data), represented all International Organization for Standardization (ISO) regions (*n*=3 Africa, *n*=17 Americas, *n*=36 Asia, *n*=39 Europe, *n*=6 Oceania and *n*=3 missing data; Fig. 2a), and *n*=10 bacterial genera, of which *n*=63/104 (61%) sequences were from *Pseudomonas* and *n*=41/104 (47%) from other Gram-negative bacilli [*Acinetobacter* (*n*=3), *Aeromonas* (*n*=1), *Citrobacter* (*n*=5), *Enterobacter* (*n*=5), *Escherichia* (*n*=6), *Klebsiella* (*n*=13), *Morganella* (*n*=1), *Raoultella* (*n*=4) and *Serratia* (*n*=3); Fig. 2b]. The overrepresentation of *Pseudomonas* spp. is consistent with previously reported *bla*_GES-5_ distribution [63]. Assembled contigs containing *bla*_GES-5_ varied dramatically in length (median=4900 bp, range=960–7 086 823 bp), reflecting the fact that the assembly of integron regions from short reads can lead to short contigs.

### Exploring *bla*_GES-5_ flanking sequence diversity with Flanker

We first used Flanker [66] for an exploratory coarse-grained clustering of the 10kbp flanking sequences both upstream and downstream of *bla*_GES-5_ (see Methods). Briefly, Flanker annotated the 104 sequences for *bla*_GES-5_ and then extracted flanking sequences in increasing 100 bp steps in both the upstream and downstream directions, up to 10 000 bp. Next, for each set of flanking sequences of a given length and direction, Flanker clustered them using Mash distances and single-linkage clustering. Fig. 3 visualizes these clusters for each contig. In addition to Flanker, we performed a secondary clustering of the cluster labels themselves (‘meta-clusters’; see Methods). In short, for both the upstream and downstream directions, we generated a matrix of contig cluster labels, calculated the Jaccard index (*JI*) of these labels (the number of shared cluster labels divided by the total number of cluster labels; see Methods) and performed a complete hierarchal clustering. This grouped contigs with similar Flanker cluster profiles, revealing distinct meta-clusters both upstream and downstream of *bla*_GES-5_, consistent with shared genetic structures in the flanking sequences. The ordering of the hierarchal clustering dendrograms provided the order of the contigs in Fig. 3.

### The genetic landscape of *bla*_GES-5_ integrons

Following the exploratory analysis with Flanker, we wanted to document the genetic structure of *bla*_GES-5_-associated integrons. To begin, we extracted flanking sequences 10 kbp upstream and downstream of the gene (using Flanker; see Methods). For all the sequences, we retrieved annotation data from the NCBI’s Prokaryotic Genome Annotation Pipeline (*n*=2/104 contigs had no annotations; see Methods). We also generated integron-specific annotations using IntegronFinder and transposase annotations using the ISfinder database (see Methods). Using IntegronFinder, we annotated for (i) integrases, (ii) *attI* and *attC* sites, (iii) P_int_ and (iv) P_c_. In total, 37 flanking sequences were annotated for an integrase, and of these, 73% (27/37) had an *attI* site and at least one *attC* site, indicating a complete integron. Notably, 22% (8/37) of integrase-positive sequences had at least one *attC* site but lacked an *attI* site, potentially consistent with the difficulty of annotating *attI* outside of class 1 integrons [45]. To predict putatively functional integrases, we included annotations for the promoters P_int_ and P_c_. Of the 37 integrases annotated, 41% (15/37) had both P_int_ and P_c_, 24% (9/37) had just P_c_ and the remaining 35% (13/37) had neither, consistent with a spectrum of putative activity.

Using annotations found between either *attI* and *attC* or an *attC* and *attC* sites, we recovered 39 putative unique gene cassettes and 195 total cassettes across the flanking sequences (Table 1). For describing integrons in this study, gene cassettes are recorded between vertical bars ‘|’, with commas separating their genes, e.g. |*a*|, |*b*, *c*| represents an integron array of two gene cassettes, one containing gene *a* and another containing genes *b* and *c*. Gene cassettes were median=1 gene long (range=1–4 genes). *bla*_GES-5_ was almost always found alone as a gene cassette (34/35), except once with an IS*Pa21* insertion sequence and *aac(6′)-Ib4* (1/35). Aminoglycoside resistance genes were found in 51% (99/195) of gene cassettes, of which most were a lone *aac(6′)-Ib4* gene (42% [42/99]). Also, observed were *dfrA*-family trimethoprim resistance genes (6/195), OXA-family beta-lactamase genes (3/195) and *ere(A*) macrolide resistance genes (2/195). Equally as common as the *aac(6′)-Ib4* cassette was a DUF1010 gene cassette (42/195), encoding an uncharacterized protein.

**Table 1.** Gene cassettes found on *bla*_GES-5_ integrons

Gene cassette	Count	Number of genes	Predicted antibiotic resistance phenotype	Median position	Nearest position	Furthest position
|*aac(6′)-Ib4*|	42	1	Aminoglycoside	2	2	3
|DUF1010|	42	1	na	3	2	3
|*bla*_GES-5_|	34	1	Carbapenem, cephalosporin and penam	1	1	2
|*aph(3′)-XV*, IS*Pa21*|	19	2	Aminoglycoside	4	4	4
|*aph(3′)-XV*|	7	1	Aminoglycoside	4	4	4
|*aac(6′)-Ib*|	6	1	Aminoglycoside	2	1	2
|*aadB*|	4	1	Aminoglycoside	2	2	2
|*aac(6′)-Ib′*|	3	1	Aminoglycoside	3	1	4
|*aac(6′)-Ib3*|	3	1	Aminoglycoside	1	1	1
|*dfrA7*|	3	1	Trimethoprim	2	2	2
|*dfrA21*|	2	1	Trimethoprim	3	1	4
|*ere(A)*|	2	1	Macrolide	na	na	na
|hypothetical protein|	2	1	na	3	3	3
|*aac(6′)−29*|	1	1	Aminoglycoside	na	na	na
|*aac(6′)−31*|	1	1	Aminoglycoside	2	2	2
|*aac(6′)-Ib*|	1	1	Aminoglycoside	na	na	na
|*aac(6′)-Ib*, IS*6100*, *repB*, *mobB*|	1	4	Aminoglycoside	2	2	2
|*aac(6′)-Il*|	1	1	Aminoglycoside	3	3	3
|*aac(6′)*|	1	1	Aminoglycoside	na	na	na
|*aadA*|	1	1	Aminoglycoside	3	3	3
|*aadA1*|	1	1	Aminoglycoside	4	4	4
|*aac(6′)-Ia*, DUF3788|	1	2	Aminoglycoside	2	2	2
|*aac(6′)-Iq*|	1	1	Aminoglycoside	1	1	1
|*aac(6′)-IIa*|	1	1	Aminoglycoside	2	2	2
|*aadA4*|	1	1	Aminoglycoside	4	4	4
|*aadA5*|	1	1	Aminoglycoside	4	4	4
|*bla*_GES-5_, IS*Pa21*, *aac(6')-Ib4*|	1	3	Carbapenem, cephalosporin, penam and aminoglycoside	1	1	1
|*dfrA5*|	1	1	Trimethoprim	na	na	na
|DM13 |	1	1	na	2	2	2
|*aac(6′)-Ib-cr9*|	1	1	Aminoglycoside	1	1	1
|hypothetical protein, *mobA*/*mobL*|	1	2	na	na	na	na
|mobilization protein|	1	1	na	na	na	na
|*bla*_OXA-17_|	1	1	Carbapenem, cephalosporin and penam	3	3	3
|*bla*_OXA-1_|	1	1	Carbapenem, cephalosporin and penam	na	na	na
|*bla*_OXA-2_|	1	1	Carbapenem, cephalosporin and penam	5	5	5
|*qac*|	1	1	na	6	6	6
|*qacG2*|	1	1	na	3	3	3
|*qacF*|	1	1	na	na	na	na
|*qnrVC4*|	1	1	Fluoroquinolone	na	na	na

From left to right, the columns report the gene cassette, count in the dataset, number of genes in the casette, predicted AMR phenotype and median, nearest and furthest position from the integrase. When a gene cassette position could not be ascertained (for example, because it was found without an integrase upstream), na is recorded. Rows coloured grey indicate those containing *bla*_GES-5_.

In terms of insertion sequences, IS*Pa21* (of the IS*110* family, the IS*1111* group) was observed 18 and 1 times in an |*aph(3′)-XV*, IS*Pa21*| and |*bla*_GES-5_, IS*Pa21*, *aac(6′)-Ib4*| gene cassette, respectively. The IS*1111* group is well documented as targeting *attC* sites in class 1 integrons [67,68]. There was also one instance of IS*1600* (of the IS*21* family) in an |*aac(6′)-Ib*, IS*6100*, *repB*, *mobB*| gene cassette. However, the terminal attachment site overlapped with a putative mobility protein, so was potentially a false positive.

We also examined the position of the gene cassettes downstream of the integrase (Table 1). To do this, we took the 30/104 contigs with an integrase and/or *attI* site and at least one gene cassette (as described above). Then, we recorded the position of the gene cassettes downstream of the integrase and *attI* site. The position of 10/40 gene cassettes could not be determined as the contig(s) contained no integrase/*attI* site. Notably, *bla*_GES-5_ gene cassettes were almost always in the first position [93% (26/28), where possible to calculate], consistent with the highest expression [69,70]. Overall, this approach is likely to underestimate the true number of gene cassettes in the sample due to difficulty annotating *attI* and *attC* sites, as well as fragmented contigs.

### Clustering *bla*_GES-5_ flanking sequences with PanGraph

Whilst the Flanker analysis captured some genetic similarity between the flanking sequences, it is order dependent: it can only compare sequence windows found in the same position upstream or downstream. In contrast, pangenome graph-based methods summarize the sharing of genes or genetic structures within a collection of sequences, independent of order. For the *bla*_GES-5_ flanking sequences, we generated a pangenome graph using PanGraph [48] (Fig. S2; see Methods). PanGraph represents each flanking sequence as a path through a graph where each node is an annotation-free multiple sequence alignment (or ‘block’), and each edge is an inferred homology breakpoint. Stretches of sequence are collapsed into blocks by evaluating the costs of both within-block diversity and splitting a block. From the 104 10 kbp *bla*_GES-5_ flanking sequences, we extracted 164 unique homology blocks, with median length=623 bp (range=99–9247 bp). The graph totalled 247 747 bp in length, of which 62% (153 992/247 747 bp) comprised unique blocks (seen only in a single flanking sequence), consistent with substantial genetic diversity in *bla*_GES-5_ flanking sequences. The 104 flanking sequences were represented by 80 unique paths in the pangenome graph (median=1 flank per unique path, range=1–6).

Next, we clustered the flanking sequences by their putative gene cassette contents. First, for the 69/80 flanking sequences containing either an *attI* and at least one *attC* site or at least two *attC* sites, we collated a list of PanGraph blocks found between the maximally upstream and downstream sites. Then, for all the flanking sequences, we generated a presence/absence matrix of these blocks. This aimed to capture the gene cassette contents as blocks, even when *attI*/*attC* sites were poorly annotated, as well as intergenic sequences. Using the presence/absence matrix, we calculated the pairwise *JI* of blocks between flanking sequences. Importantly, *JI* is indifferent to the ordering of blocks, such as those corresponding to shuffled gene cassettes from identical integrons. The resulting distance matrix was used for complete-linkage hierarchical clustering to order the flanking sequences (Figs 4a and S3 presents a zoomed-in view of only the putative integron sequences, including annotations for the gene cassettes). We chose complete-linkage hierarchal clustering because it is robust to cluster shape, which we *a priori* did not know, and less sensitive to outliers (for example, contigs with unique blocks) than other clustering approaches.

### The lifestyles of *bla*_GES-5_ integrons

Integrons can be found on both chromosomes and plasmids, so they can have different ‘lifestyles’ of transmission: either vertically on the chromosome or horizontally on plasmids, potentially moving between species. The inactivation of chromosomal integron integrases can fix a resistance phenotype within a strain [71]. In contrast, for conjugative plasmids, their transfer into a new cell can trigger an SOS response, which has been shown to upregulate integrase activity, leading to gene cassette gain, loss and shuffling [72]. The genomic context of an integron is therefore crucial for understanding its epidemiology and evolution. To explore the lifestyles of *bla*_GES-5_ integrons, we compared their genomic contexts and sampling geography and year to their genetic structure.

We first assigned the *bla*_GES-5_ contigs as chromosomal or plasmid if a sequence type (ST) or replicon type could be identified (Fig. 4e; see Methods). In total, 7% (7/107) contigs were classified as chromosomal (all *P. aeruginosa; n*=5 ST235, *n*=1 ST316 and *n*=1 ST654), 20% (21/104) plasmid (*n*=5 IncP6, *n*=5 IncQ2, *n*=3 IncQ1, *n*=1 IncL/M and *n*=7 from untypeable replicon clusters) and 73% (76/104) neither, reflecting the short length of most of the contigs. Of the plasmids, 71% (15/21) were predicted to be mobilizable (19% [4/21] non-mobilizable and 10% [2/21] conjugative). In total, six contigs were assigned both an ST and contained putative plasmid replicons (all *P. aeruginosa*, *n*=5 ST235 and *n*=1 ST316). For ST235, 5/5 contained an IncP replicon, as well as ‘rep_cluster_10’ and ‘rep_cluster_80’. One ST235 contig also contained ‘rep_cluster_322’. This is consistent with the identification of previously described genomic islands in ST235 [73]. In addition to classifying the contigs by putative origin, we also integrated the NCBI sampling metadata from earlier: isolate genus (Fig. 4f), sample ISO region (Fig. 4g) and sampling year (Fig. 4h; see Methods).

The PanGraph-based clustering revealed a family of 42 class 1 integrons, of which 2/42 were from *P. aeruginosa* ST235, 1/42 contained ‘rep_cluster_80’ and 39/42 were from unclassified contigs. The integron demonstrated a well-conserved gene cassette array: whilst only 2/42 carried |*bla*_GES-5_|*aac(6′)-Ib4*|DUF1010|*aph(3′)−XV* |IS*Pa21*| with complete *attC* and *attI* boundaries, a total of 30/42 carried these genes in the same order. Moreover, 6/42 terminated before *ISPa21* with an *attC* site and 3/42 without. Most commonly, *ISPa21* inserted within an |*aph(3′)−XV*| cassette to form an |*aph(3′)−XV*, IS*Pa21*| cassette (16/42) but also once between |*bla*_GES-5_| and |*aac(6′)-Ib4*| to form a |*bla*_GES-5_, IS*Pa21*, *aac(6′-Ib4*| cassette (1/42). Of the annotated promoters, 50% (5/10) had both P_int_ and P_c_, 20% (2/10) had no P_int_ or P_c_ and 10% (1/10) had P_c_ but no P_int_.

In contrast to the ST235-associated integrons, the 21 plasmid-associated integrons were more varied, representing both *intI1* (10/21) and *intI3* (5/21; 6/21 broke off before the integrase). Within plasmid families, integron contents varied dramatically. Taking the largest identified replicon family, *n*=5 IncP6 plasmids, and gene cassettes with complete *attI*/*attC* boundaries, *n*=1/5 contained |*bla*_GES-5_|, *n*=1/5 |*bla*_GES-5_|, |*aac(6′)−31*|, |*qacG2*|, |*aadA5*|, *n*=1/5 |*bla*_GES-5_|, |*aac(6′)-IIa*|, |*aadA*|, *n*=1/5 |*bla*_GES-5_|, |*aac(6′)-Ia*, DUF3788| and *n*=1/5 |*bla*_GES-5_|, |*aac(6′)-Ib4*|, |*qacF*|. No plasmids of the same replicon type shared the same gene cassette array.

### Integron SNV profiles are unreliable epidemiological markers

SNV profiling of the integrase and gene cassettes might help track integrons. However, there are potential pitfalls. Integrases are known to commonly undergo indel events, which can inactivate the gene and fix the gene cassettes in place [30]. Moreover, integrons can undergo frequent changes in cassette array, for instance, under intermittent antimicrobial exposure [70], meaning that it might not be possible to consistently track specific gene cassette-integrase pairings. Using the curated dataset of *bla*_GES-5_ integron sequences, we explored SNV profiling of both the integrase and *bla*_GES-5_ gene.

For the integrase genes, we produced multiple-sequence alignments of the 37 integrase annotations found in the flanking sequences (see Methods). Based on complete-linkage clustering of pairwise SNV distances, this revealed two distinct clusters of integrases (Fig. S4). Cluster 1 (C1) contained 29 *intI1* integrases, median=1 SNP apart (range=0–3 SNPs), and were all 1 014 bp in length except 2 at 867 bp and 924 bp, consistent with minimal indel events. Cluster 2 (C2) contained eight *intI3* integrases, median=1.5 SNPs apart (range=0–14 SNPs), and were more variable in length (*n*=1 816 bp, *n*=1 897 bp, *n*=1 990 bp, *n*=2 1,017 bp, *n*=3 1,041 bp), consistent with a higher diversity of indels. The integrase gene (*intI1* or *intI3*) is shown by colour in Fig. 4a (blue or green, respectively). Additionally, *attI* and *attC* sites are shown in orange and red, respectively, and for clarity, the presence/absence of promoters P_int_/P_c_ is indicated in Fig. 4b (see Methods).

For C1, we removed the two shorter integrases, aligned the remainder and determined the SNV sites (see Methods). This recovered five SNV profiles of length four nucleotides (positions 87, 90, 95 and 115): *n*=12 CTGA, *n*=6 CCGC, *n*=5 CTGC, *n*=3 CCCC and *n*=1 TCGC. The comparison of C1 SNV profiles against flanking sequence structure is shown in Fig. 4c. In one case, a duplicated flanking sequence represented two SNV profiles (CTGA/CTGC).

Integrase SNV profiles showed some concordance with the resistance profile and wider flanking structure (see Fig. S5 for *intI1* SNV profiles against flank block contents). For example, *intI1* profile CCGC was always found downstream of a conserved structure, containing *merA* (mercuric reductase: *n*=5/6 sequences, *n*=1/6 times the sequence did not continue upstream of *intI1*), which were also the only instances of the *merA* operon in the dataset. Moreover, all instances (6/6) of profile CCGC had P_c_ but no P_int_. Downstream of profile CCGC, the overall gene cassette array was more varied: *n*=3/6 carried |*bla*_GES-5_|, |*aadB*|, *n*=2/6 carried |*bla*_GES-5_| and *n*=1/6 |*bla*_GES-5_|, |*aadB*|, |*aac(6′)-Il*|, |*aadA4*|. Alternatively, *intI1* profile CCCC was found with a variety of flanking structures both upstream (*n*=2/3 included *repB* and the toxin-antitoxin genes *brnT*/*brnA*; *n*=1/3 included the restriction enzyme *eco57IR*) and downstream (gene cassette arrays: *n*=1/3 |*bla*_GES-5_|, |*aac(6')−31*|, |*qacG2*|, |*aadA5*|, *n*=1/3 |*bla*_GES-5_|, *n*=1/3 |*bla*_GES-5_, *aac(6′)-IIa*, *aadA*|). However, all instances (3/3) of profile CCCC had both P_c_ and P_int_. No SNV profile was consistently found with the same upstream/downstream structure or gene cassette array. Moreover, the integrase SNV profile did not always predict promoter presence: for instance, profile CTGA had both P_c_ and P_int_ 58% (7/12) of the time, missing P_int_ 17% (2/12), and had neither 25% (3/12). P_int_ can fall downstream of the integrase [8], potentially causing an inconsistency between the *intI1* SNV profile and promoter presence.

For *bla*_GES-5_, we similarly produced multiple-sequence alignments of the 104 annotations (all were 864 bp long and encoded identical proteins). This revealed nine sSNV profiles of length 12 nucleotides (positions 15, 97, 126, 171, 177, 195, 292, 494, 504, 696, 759 and 837), of which one represented 85% (88/104) of sequences (*n*=88 CCGGTGGGACAA, *n*=4 CCGGCGGGACAA, *n*=4 CCCGTGGGACAA, *n*=2 CCGGTGGGACAG, *n*=2 CCGGTGGGCCA, *n*=1 CCGGTGGGACCA, *n*=1 CCGGTGGGATAA, *n*=1 TCGGTGGGACAA and *n*=1 CCGATGGGACAA). The comparison of *bla*_GES-5_ sSNV profiles against flanking sequence structure is shown in Fig. 4d. For the minority sSNV profiles, it was challenging to evaluate their utility as epidemiological markers; however, none consistently represented the same flanking structures. Of possible interest was sSNV profile CCGGCGGGACAA, which was always associated with integrase SNV profile CCGC and *Pseudomonas* spp., with 1/4 contigs assigned chromosomal and the remainder unclassifiable. Overall, across the *bla*_GES-5_-associated integrons, there are globally dominant *intI1* (CTGA) and *bla*_GES-5_ (CCGGTGGGACAA) SNVs. This diminishes the epidemiological value of using these SNVs on a global scale. Moreover, if these common variants are present in local contexts, such as in hospital or community surveillance, it might also be limiting.

## Discussion

Class 1 integrons play a crucial role in global AMR spread, yet they can be challenging to analyse and track. Traditional reference-based methods have struggled to keep up with the rapid increase in integron diversity identified in sequencing data, as well as the challenges posed by fragmented assemblies, particularly those constructed from short-read data. To address these issues, we have presented a novel pangenome graph-based workflow that effectively utilizes truncated integron contigs and is robust to gene cassette shuffling. The utility of our approach is exemplified by the integron-associated carbapenemase gene *bla*_GES-5_, originally identified in 2004 on a class 1 integron [74]. To our knowledge, this is the first review of *bla*_GES-5_ global genomic epidemiology using all public *bla*_GES-5_-containing contigs and indeed of any GES variant.

We first curated a global dataset of 104 representative *bla*_GES-5_ contigs from the NCBI, spanning more than 15 years. These contained 39 unique gene cassettes, arranged in 17 unique complete arrays (namely those that started with either an integrase or an *attI* site, and contained at least one complete gene cassette; see Fig. S3). Many more arrays were likely sampled, but we were limited by missing gene and *attI* and *attC* site annotations, as well as ‘broken’ contigs. At the time of writing, the INTEGRALL database held just nine unique gene cassette arrays for *bla*_GES-5_ integrons, all of which were class 1 integrons [14]. In contrast, we identified eight *bla*_GES-5_ contigs containing class 3 integron integrases, consistent with *bla*_GES-5_ transfer events between integron classes.

We then applied Flanker to the dataset of *bla*_GES-5_ contigs, revealing shared genetic structures both upstream and downstream of the gene in an exploratory analysis. Next, we constructed a pangenome graph for the dataset with PanGraph and used the shared ‘blocks’ of sequences to cluster the contigs. This approach seemed robust to both variable gene cassette synteny and the inclusion of short or incomplete contigs. Our findings suggested different lifestyles for integrons found on the chromosome versus plasmids, where the former is well conserved with structural variants evolving from interactions from parasitic transposable elements, and the latter is more structurally diverse. Lastly, we examined the potential of SNV profiling for integron epidemiology, first proposed in Partridge *et al*. [3]. Overall, the utility of SNV profiling for *intI1* integrases and *bla*_GES-5_ gene cassettes remains unclear on a global scale and perhaps better suited to localized geographies whereby invading integrons can be better distinguished from native integrons.

Our analysis has limitations. *bla*_GES-5_ gene cassettes were usually found in the first position, immediately downstream of the integrase. Yet, relying solely on sequence data meant that it was impossible to determine whether this meant higher expression of the gene. Moreover, identifying a gene cassette in the first position might also indicate that it was the most recently acquired [75], but this could not be explored here since the gene cassettes might have shuffled since a capture event. Also, by only selecting contigs with *bla*_GES-5_ combined with the fragmentary nature of integron sequencing, it is possible that our methodology favoured selecting integrons with *bla*_GES-5_ in the first position. Overall, this made it challenging to draw meaningful inferences about the evolutionary history of *bla*_GES-5_. Additionally, since we constructed our dataset from the NCBI, it was susceptible to its clinical and taxonomic biases. Therefore, it might disproportionately represent studies that selectively sampled for carbapenemase-producing isolates and/or isolates from patients on cephalosporins/carbapenems, which favours the *bla*_GES-5_ phenotype and hence close proximity to the promoter. In a non-clinical sample, *bla*_GES-5_ might be more varied in position.

There were also challenges when determining the putative activity of the integrons. Our approach relied on annotations for the integrase promoters P_c_ and P_int_ and therefore hinged on a reference database. Although not feasible here, integrase functionality should be experimentally validated. This might explain that whilst both promoters were annotated within * P. aeruginosa* ST235 integrons, the order of the gene cassettes remained mostly static. Conversely, the conservation of these integrons might have been influenced by the IS*Pa21* insertions, which might disrupt *attC* sites and recombination activity. Currently, limited experimental evidence exists on the impact of IS*1111*-group insertion sequences on integron function and gene cassette expression. Furthermore, when evaluating integron function, we also did not investigate the distribution of Shine–Dalgarno sequences in the *attI* sites, which might contribute to gene cassette expression [76].

It was also challenging to classify the contigs as plasmid or chromosomal. Many contigs were extremely short and could not be typed because the discriminating information was not present: chromosomal housekeeping genes or plasmid replicon genes. Ultimately, if the information is not present in a contig, it cannot be classified with full confidence. Also, due to chromosomal genomic islands, it is possible that some plasmid classifications were false positives, for example, in *P. aeruginosa* ST235, often contains a genomic island known as GI2, which is known to house *bla*_GES-5_ [73]. Compared with acquiring a plasmid-borne ‘mobile integron’, this might be an important pathway for long-term integron acquisition within a strain.

Additionally, PanGraph was developed for complete genomes, so it is calibrated for long homologous blocks. Passing in fragmented short contigs is not its intended use, so the higher proportion of short homologous blocks could produce unexpected results. This was not investigated here but should be explored more systematically in further studies.

Although not performed here, the simultaneous analysis of the flanking sequences of other GES variants might be appropriate, considering how closely related many are (Fig. S1a). Within *bla*_GES-5_ alone, we identified 12 synonymous SNV sites, which might alter phenotype or facilitate the evolution between GES variants as ‘bridging genes’. Hence, a genomic or genetic context for one GES variant might indeed be found for another. However, incorporating this extra axis was out of the scope of this analysis.

In summary, we have presented a global analysis of *bla*_GES-5_ genetic contexts, including an approach to NCBI dataset curation, followed by exploratory and in-depth analysis. Whilst many contigs were too short to be classified as chromosomal or plasmid, a pangenome graph approach was able to give insights into the integrons involved in globally disseminating *bla*_GES-5_. Chromosomal *P. aeruginosa* ST235-associated integrons were highly conserved, whilst plasmid IncP6 integrons were diverse. This suggests that reference-based approaches are more suitable for the former, whilst flexible graph-based approaches better suit the latter. Future studies should seek to better characterize the mechanisms and dynamics driving these distinct lifestyles of *bla*_GES-5_-associated integrons.

## supplementary material

10.1099/mgen.0.001312 Fig. S1.

10.1099/mgen.0.001312 Table S1.

## Abbreviations

AMR: antimicrobial resistance
ISO: International Organization for Standardization
*JI*: Jaccard index
MGE: mobile genetic element
MicroBIGG-E: Microbial Browser for Identification of Genetic and Genomic Elements
SNV: single-nucleotide variant
sSNV: synonymous single-nucleotide variant
ST: sequence type

## References

[1] Baquero F, Martínez JL, F Lanza V, Rodríguez-Beltrán J, Galán JC (2021). Evolutionary pathways and trajectories in antibiotic resistance. Clin Microbiol Rev.

[2] Sheppard AE, Stoesser N, Wilson DJ, Sebra R, Kasarskis A (2016). Nested Russian doll-like genetic mobility drives rapid dissemination of the carbapenem resistance gene *bla*KPC. Antimicrob Agents Chemother.

[3] Partridge SR, Tsafnat G, Coiera E, Iredell JR (2009). Gene cassettes and cassette arrays in mobile resistance integrons. FEMS Microbiol Rev.

[4] Nunes-Düby SE, Kwon HJ, Tirumalai RS, Ellenberger T, Landy A (1998). Similarities and differences among 105 members of the Int family of site-specific recombinases. Nucleic Acids Res.

[5] Messier N, Roy PH (2001). Integron integrases possess a unique additional domain necessary for activity. J Bacteriol.

[6] Demarre G, Frumerie C, Gopaul DN, Mazel D (2007). Identification of key structural determinants of the IntI1 integron integrase that influence attC x attI1 recombination efficiency. Nucleic Acids Res.

[7] Jové T, Da Re S, Denis F, Mazel D, Ploy M-C (2010). Inverse correlation between promoter strength and excision activity in class 1 integrons. PLoS Genet.

[8] Escudero JA, Loot C, Nivina A, Mazel D (2015). The integron: adaptation on demand. Microbiol Spectr.

[9] Kaushik M, Kumar S, Kapoor RK, Virdi JS, Gulati P (2018). Integrons in *Enterobacteriaceae*: diversity, distribution and epidemiology. Int J Antimicrob Agents.

[10] Gillings M, Boucher Y, Labbate M, Holmes A, Krishnan S (2008). The evolution of class 1 integrons and the rise of antibiotic resistance. J Bacteriol.

[11] Ghaly TM, Chow L, Asher AJ, Waldron LS, Gillings MR (2017). Evolution of class 1 integrons: mobilization and dispersal via food-borne bacteria. PLoS One.

[12] Deng Y, Bao X, Ji L, Chen L, Liu J (2015). Resistance integrons: class 1, 2 and 3 integrons. Ann Clin Microbiol Antimicrob.

[13] Gillings MR (2017). Class 1 integrons as invasive species. Curr Opin Microbiol.

[14] Moura A, Soares M, Pereira C, Leitão N, Henriques I (2009). INTEGRALL: a database and search engine for integrons, integrases and gene cassettes. Bioinformatics.

[15] Gillings MR, Gaze WH, Pruden A, Smalla K, Tiedje JM (2015). Using the class 1 integron-integrase gene as a proxy for anthropogenic pollution. ISME J.

[16] Ross K, Varani AM, Snesrud E, Huang H, Alvarenga DO (2021). TnCentral: a prokaryotic transposable element database and web portal for transposon analysis. *mBio*.

[17] Sheppard AE, Stoesser N, German-Mesner I, Vegesana K, Sarah Walker A (2018). TETyper: a bioinformatic pipeline for classifying variation and genetic contexts of transposable elements from short-read whole-genome sequencing data. Microb Genom.

[18] Johansson MHK, Bortolaia V, Tansirichaiya S, Aarestrup FM, Roberts AP (2021). Detection of mobile genetic elements associated with antibiotic resistance in *Salmonella enterica* using a newly developed web tool: MobileElementFinder. J Antimicrob Chemother.

[19] Carattoli A, Zankari E, García-Fernández A, Voldby Larsen M, Lund O (2014). *In silico* detection and typing of plasmids using PlasmidFinder and plasmid multilocus sequence typing. Antimicrob Agents Chemother.

[20] Arredondo-Alonso S, Gladstone RA, Pöntinen AK, Gama JA, Schürch AC (2023). Mge-cluster: a reference-free approach for typing bacterial plasmids. *NAR Genom Bioinform*.

[21] Redondo-Salvo S, Bartomeus-Peñalver R, Vielva L, Tagg KA, Webb HE (2021). COPLA, a taxonomic classifier of plasmids. BMC Bioinform.

[22] Jolley KA, Bray JE, Maiden MCJ (2018). Open-access bacterial population genomics: BIGSdb software, the PubMLST.org website and their applications. Wellcome Open Res.

[23] Katoh K, Standley DM (2013). MAFFT multiple sequence alignment software version 7: improvements in performance and usability. Mol Biol Evol.

[24] Ghaly TM, Penesyan A, Pritchard A, Qi Q, Rajabal V (2022). Methods for the targeted sequencing and analysis of integrons and their gene cassettes from complex microbial communities. Microb Genom.

[25] Buongermino Pereira M, Österlund T, Eriksson KM, Backhaus T, Axelson-Fisk M (2020). A comprehensive survey of integron-associated genes present in metagenomes. BMC Genom.

[26] Abramova A, Karkman A, Bengtsson-Palme J (2024). Metagenomic assemblies tend to break around antibiotic resistance genes. BMC Genomics.

[27] Zhang AN, Li L-G, Ma L, Gillings MR, Tiedje JM (2018). Conserved phylogenetic distribution and limited antibiotic resistance of class 1 integrons revealed by assessing the bacterial genome and plasmid collection. Microbiome.

[28] Mazel D (2006). Integrons: agents of bacterial evolution. Nat Rev Microbiol.

[29] Boucher Y, Labbate M, Koenig JE, Stokes HW (2007). Integrons: mobilizable platforms that promote genetic diversity in bacteria. Trends Microbiol.

[30] Cambray G, Sanchez-Alberola N, Campoy S, Guerin É, Da Re S (2011). Prevalence of SOS-mediated control of integron integrase expression as an adaptive trait of chromosomal and mobile integrons. Mob DNA.

[31] Ghaly TM, Tetu SG, Gillings MR (2021). Predicting the taxonomic and environmental sources of integron gene cassettes using structural and sequence homology of attC sites. *Commun Biol*.

[32] Macesic N, Hawkey J, Vezina B, Wisniewski JA, Cottingham H (2023). Genomic dissection of endemic carbapenem resistance reveals metallo-beta-lactamase dissemination through clonal, plasmid and integron transfer. Nat Commun.

[33] Yang D-D, Rusch LM, Widney KA, Morgenthaler AB, Copley SD (2024). Synonymous edits in the *Escherichia coli* genome have substantial and condition-dependent effects on fitness. Proc Natl Acad Sci U S A.

[34] Zwart MP, Schenk MF, Hwang S, Koopmanschap B, de Lange N (2018). Unraveling the causes of adaptive benefits of synonymous mutations in TEM-1 β-lactamase. Heredity.

[35] Ballard A, Bieniek S, Carlini DB (2019). The fitness consequences of synonymous mutations in *Escherichia coli*: experimental evidence for a pleiotropic effect of translational selection. Gene.

[36] Sievers F, Higgins DG (2018). Clustal Omega for making accurate alignments of many protein sequences. Protein Sci.

[37] Naas T, Oueslati S, Bonnin RA, Dabos ML, Zavala A (2017). Beta-lactamase database (BLDB) - structure and function. J Enzyme Inhib Med Chem.

[38] Sayers EW, Bolton EE, Brister JR, Canese K, Chan J (2022). Database resources of the national center for biotechnology information. Nucleic Acids Res.

[39] Feldgarden M, Brover V, Gonzalez-Escalona N, Frye JG, Haendiges J (2021). AMRFinderPlus and the reference gene catalog facilitate examination of the genomic links among antimicrobial resistance, stress response, and virulence. Sci Rep.

[40] Camacho C, Coulouris G, Avagyan V, Ma N, Papadopoulos J (2009). BLAST+: architecture and applications. BMC Bioinform.

[41] Ondov BD, Starrett GJ, Sappington A, Kostic A, Koren S (2019). Mash Screen: high-throughput sequence containment estimation for genome discovery. Genome Biol.

[42] Cock PJA, Antao T, Chang JT, Chapman BA, Cox CJ (2009). Biopython: freely available Python tools for computational molecular biology and bioinformatics. Bioinformatics.

[43] Kans J (2014).

[44] Tatusova T, DiCuccio M, Badretdin A, Chetvernin V, Nawrocki EP (2016). NCBI prokaryotic genome annotation pipeline. Nucleic Acids Res.

[45] Néron B, Littner E, Haudiquet M, Perrin A, Cury J (2022). IntegronFinder 2.0: identification and analysis of integrons across bacteria, with a focus on antibiotic resistance in *Klebsiella*. Microorganisms.

[46] Seeman T (2015). Abricate, Github. https://github.com/tseemann/abricate.

[47] Siguier P, Perochon J, Lestrade L, Mahillon J, Chandler M (2006). ISfinder: the reference centre for bacterial insertion sequences. Nucleic Acids Res.

[48] Noll N, Molari M, Shaw LP, Neher RA (2023). *PanGraph*: scalable bacterial pan-genome graph construction. Microb Genom.

[49] Chen Y, Ye W, Zhang Y, Xu Y (2015). High speed BLASTN: an accelerated MegaBLAST search tool. Nucleic Acids Res.

[50] Seemann T (2017). mlst, Github. https://github.com/tseemann/mlst.

[51] Robertson J, Nash JHE (2018). MOB-suite: software tools for clustering, reconstruction and typing of plasmids from draft assemblies. Microb Genom.

[52] Seeman T (2021). snp-dists, Github. https://github.com/tseemann/snp-dists.

[53] Page AJ, Taylor B, Delaney AJ, Soares J, Seemann T (2016). *SNP-sites*: rapid efficient extraction of SNPs from multi-FASTA alignments. Microb Genom.

[54] Wickham H (2016). Ggplot2: Elegant Graphics for Data Analysis.

[55] Wick RR, Schultz MB, Zobel J, Holt KE (2015). Bandage: interactive visualization of de novo genome assemblies. Bioinformatics.

[56] Acman M, Wang R, van Dorp L, Shaw LP, Wang Q (2022). Role of mobile genetic elements in the global dissemination of the carbapenem resistance gene *bla*_NDM_. Nat Commun.

[57] Poirel L, Le Thomas I, Naas T, Karim A, Nordmann P (2000). Biochemical sequence analyses of GES-1, a novel class A extended-spectrum beta-lactamase, and the class 1 integron In52 from *Klebsiella pneumoniae*. Antimicrob Agents Chemother.

[58] Poirel L, Carrër A, Pitout JD, Nordmann P (2009). Integron mobilization unit as a source of mobility of antibiotic resistance genes. Antimicrob Agents Chemother.

[59] Kanayama A, Kawahara R, Yamagishi T, Goto K, Kobaru Y (2016). Successful control of an outbreak of GES-5 extended-spectrum β-lactamase-producing *Pseudomonas aeruginosa* in a long-term care facility in Japan. J Hosp Infect.

[60] Jeong SH, Bae IK, Kim D, Hong SG, Song JS (2005). First outbreak of *Klebsiella pneumoniae* clinical isolates producing GES-5 and SHV-12 extended-spectrum beta-lactamases in Korea. Antimicrob Agents Chemother.

[61] Ellington MJ, Davies F, Jauneikaite E, Hopkins KL, Turton JF (2020). A multispecies cluster of GES-5 carbapenemase-producing enterobacterales linked by a geographically disseminated plasmid. Clin Infect Dis.

[62] Chudejova K, Rotova V, Skalova A, Medvecky M, Adamkova V (2018). Emergence of sequence type 252 *Enterobacter cloacae* producing GES-5 carbapenemase in a Czech hospital. Diagn Microbiol Infect Dis.

[63] Doumith M, Alhassinah S, Alswaji A, Alzayer M, Alrashidi E (2021). Genomic characterization of carbapenem-non-susceptible *Pseudomonas aeruginosa* clinical isolates from Saudi Arabia revealed a global dissemination of GES-5-producing ST235 and VIM-2-producing ST233 sub-lineages. Front Microbiol.

[64] Pablo-Marcos D, Siller M, Agüero J, Álvarez-Justel A, García-Fernández S (2023). Are GES carbapenemases underdiagnosed? An allelic discrimination assay for their accurate detection and differentiation. J Microbiol Methods.

[65] Gonzalez C, Volland H, Oueslati S, Niol L, Legrand C (2023). Evaluation of a new rapid immunochromatographic assay for the detection of GES-producing Gram-negative bacteria. J Antimicrob Chemother.

[66] Matlock W, Lipworth S, Constantinides B, Peto TEA, Walker AS (2021). Flanker: a tool for comparative genomics of gene flanking regions. Microb Genom.

[67] Tetu SG, Holmes AJ (2008). A family of insertion sequences that impacts integrons by specific targeting of gene cassette recombination sites, the IS1111-attC Group. J Bacteriol.

[68] Post V, Hall RM (2009). Insertion sequences in the IS1111 family that target the attC recombination sites of integron-associated gene cassettes. FEMS Microbiol Lett.

[69] Collis CM, Hall RM (1995). Expression of antibiotic resistance genes in the integrated cassettes of integrons. Antimicrob Agents Chemother.

[70] Souque C, Escudero JA, MacLean RC (2021). Integron activity accelerates the evolution of antibiotic resistance. eLife.

[71] Gillings MR, Holley MP, Stokes HW, Holmes AJ (2005). Integrons in Xanthomonas: a source of species genome diversity. Proc Natl Acad Sci U S A.

[72] Baharoglu Z, Bikard D, Mazel D (2010). Conjugative DNA transfer induces the bacterial SOS response and promotes antibiotic resistance development through integron activation. PLoS Genet.

[73] Roy Chowdhury P, Scott M, Worden P, Huntington P, Hudson B (2016). Genomic islands 1 and 2 play key roles in the evolution of extensively drug-resistant ST235 isolates of *Pseudomonas aeruginosa*. Open Biol.

[74] Vourli S, Giakkoupi P, Miriagou V, Tzelepi E, Vatopoulos AC (2004). Novel GES/IBC extended-spectrum beta-lactamase variants with carbapenemase activity in clinical enterobacteria. FEMS Microbiol Lett.

[75] Gillings MR (2014). Integrons: past, present, and future. Microbiol Mol Biol Rev.

[76] Papagiannitsis CC, Tzouvelekis LS, Tzelepi E, Miriagou V (2017). *attI1*-located small open reading frames ORF-17 and ORF-11 in a class 1 integron affect expression of a gene cassette possessing a canonical shine-dalgarno sequence. Antimicrob Agents Chemother.

