# Supplementary material for: Global genomic epidemiology of blaGES-5 carbapenemase-associated integrons
Source: Microb Genom. 2024 Dec 4;10(12):001312. doi: 10.1099/mgen.0.001312 (PMC11616780; doi:10.1099/mgen.0.001312)
Supplement: Fig. S1. [file mgen-10-01312-s001.pdf]

Supplementary materials

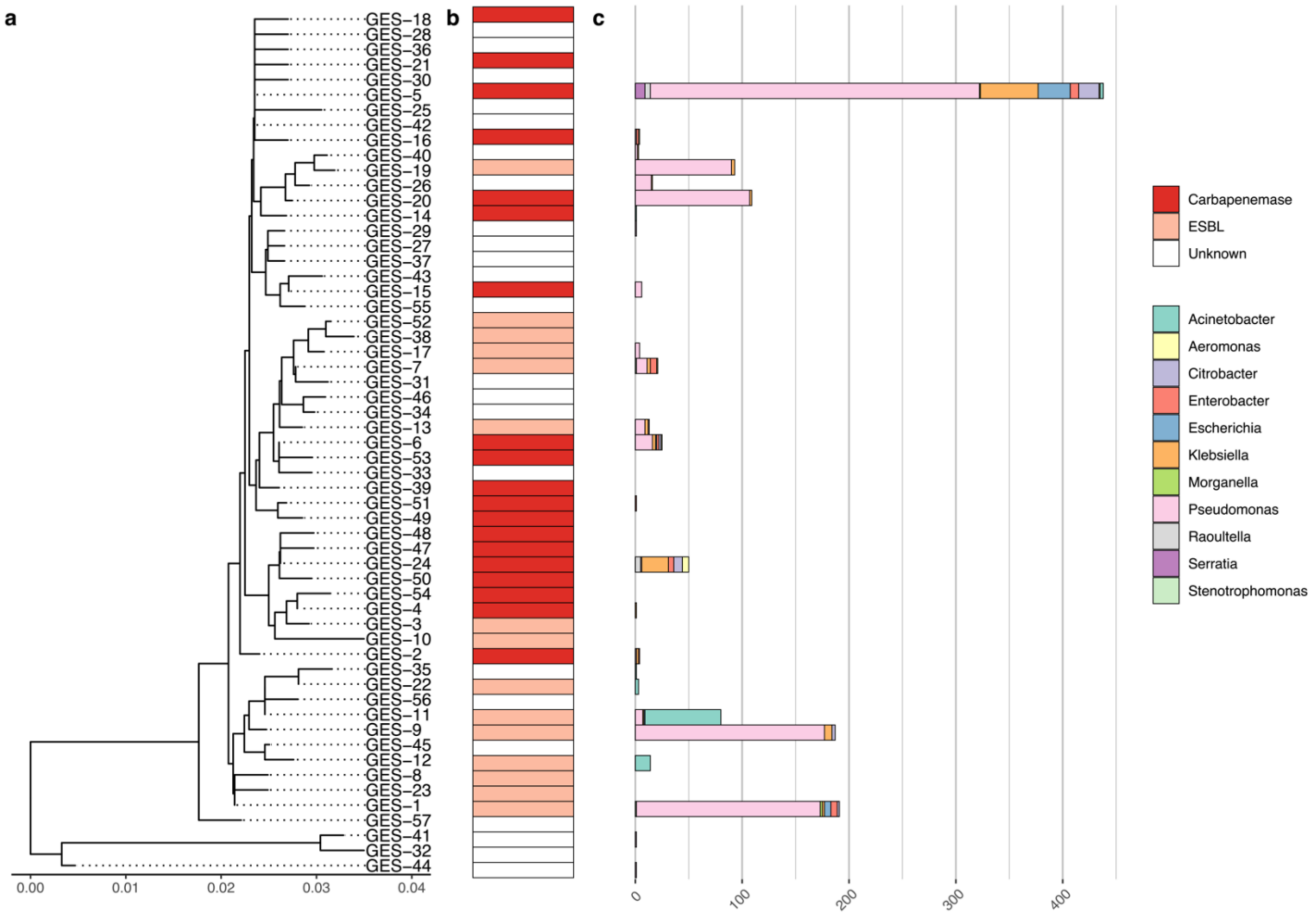

Figure S1. Distribution of NCBI GES variants.

(a) Midpoint rooted phylogeny of *n*=57 GES variants, the *x*-axis represents the number of nucleotide substitutions per site . **(b)** Experimentally determined hydrolytic profile[37]. 'Unknown' represents missing data. **(c)** Genera distribution of NCBI contig annotations**.**

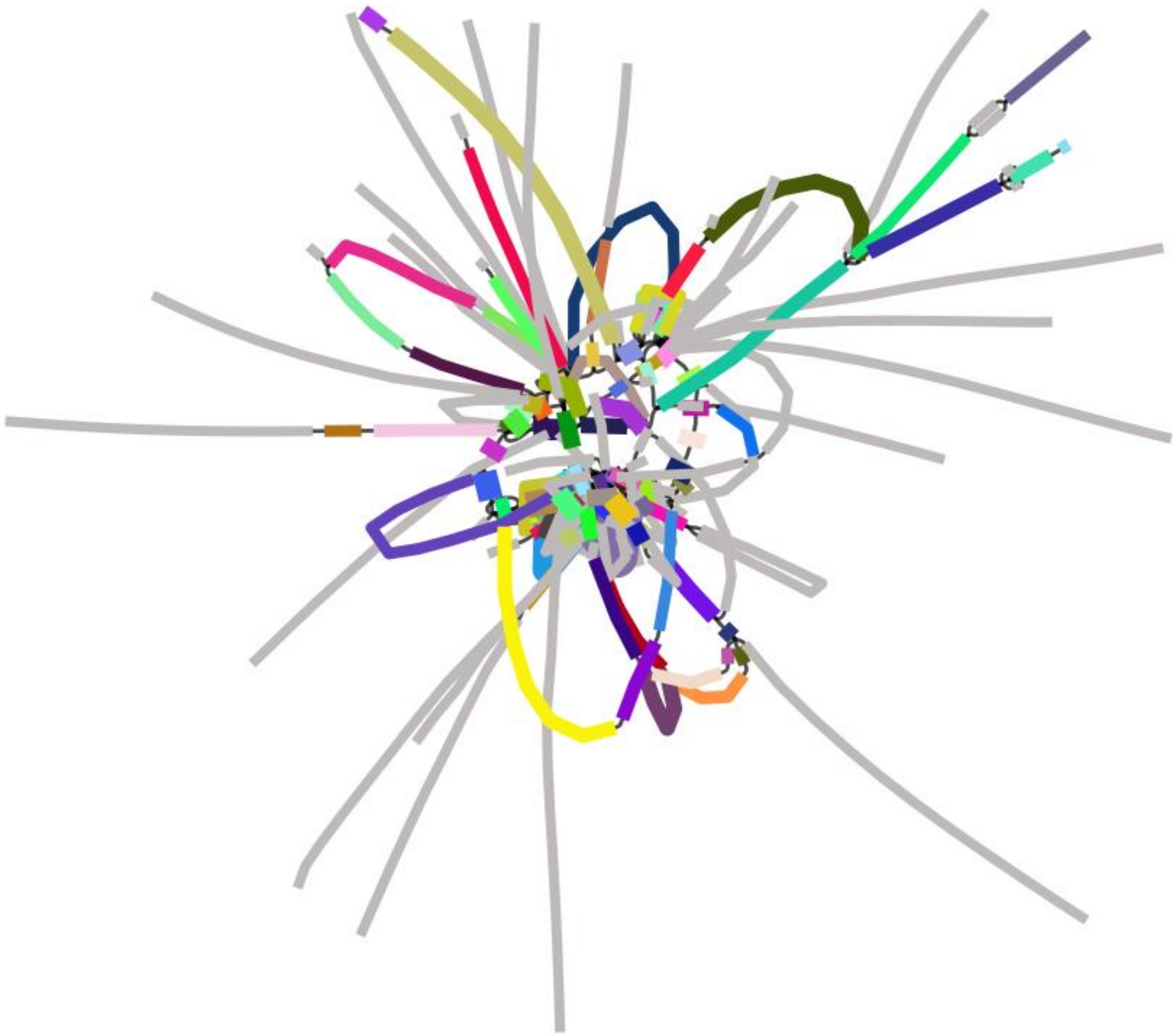

Figure S2. ***bla*$_{\mathbf{GES\text{-}5}}$ 10,000bp flanking sequence PanGraph visualised in Bandage.**

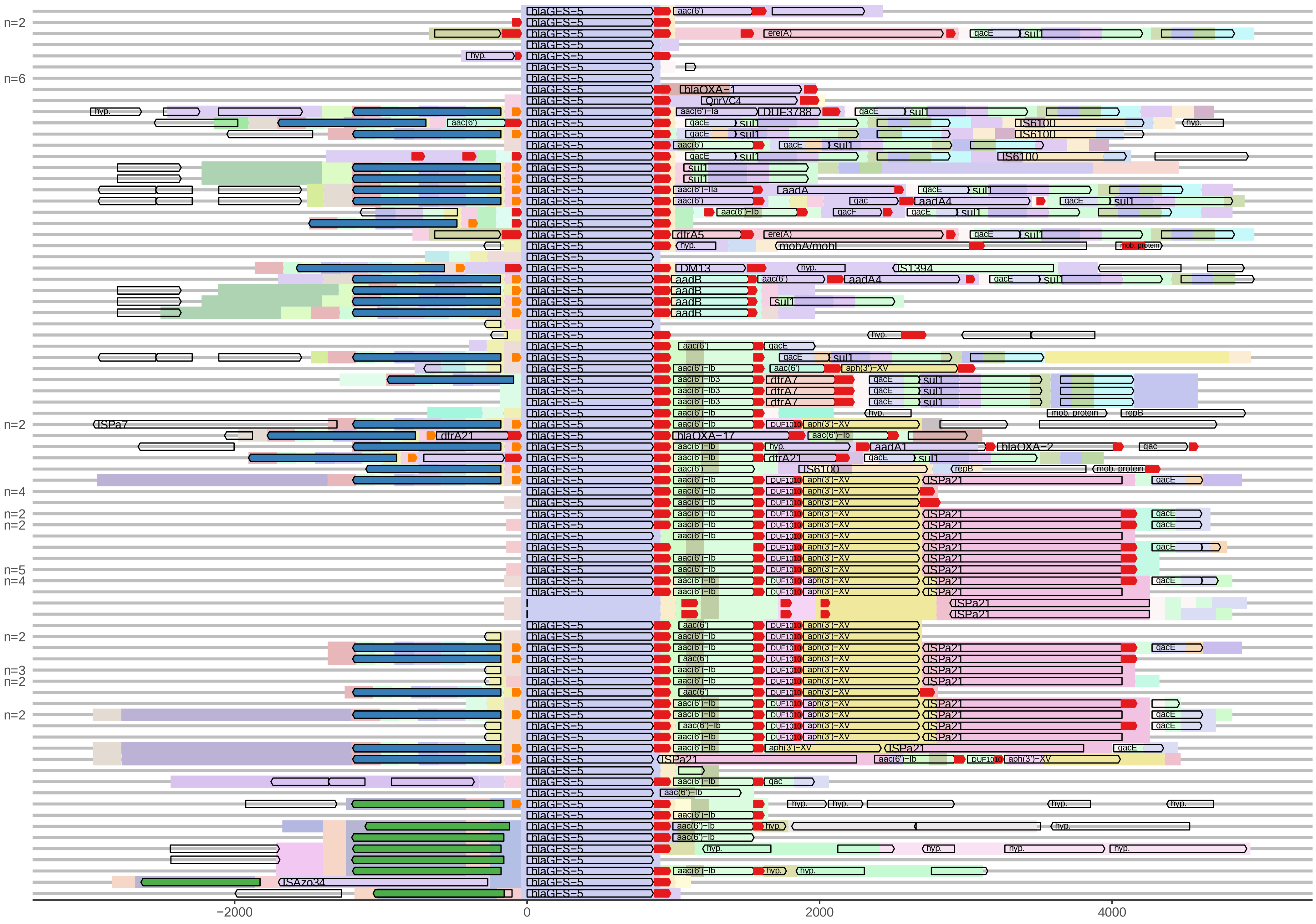

n=2
n=6
n=4
n=5
n=3
blaGES-5
aac(6')-Ib
aac(6')-Ib3
aac(6')-IIa
aac(6')-Ia
aac(6')
aph(3')-XV
DUF1010
ISPa21
ISPa7
IS6100
IS1394
ISAzo34
qacE
sul1
dfrA1
dfrA5
dfrA7
dfrA21
aadA1
aadA4
aadB
blaOXA-1
blaOXA-2
blaOXA-17
ere(A)
repB
mob. protein
hyp.
QnrVC4
DUF3788
mobA/mobL
-2000
0
2000
4000

Figure S3. Gene cassette annotations for *bla*$_{GES-5}$-associated integrons.

Flanking sequences are ordered as in Figure 4a but zoomed-in and overlayed with CDS annotations (black outlined arrows) and integron-specific annotations: *intI1* in blue, *intI3* in green, *attI* in orange, and *attC* in red. Numbers to the left indicate duplicates as in Figure 4a. Gene names are provided for any annotation found between any two *attI*/*attC* sites, as well as for 3'-conserved genes *qacE* and *sul1*.

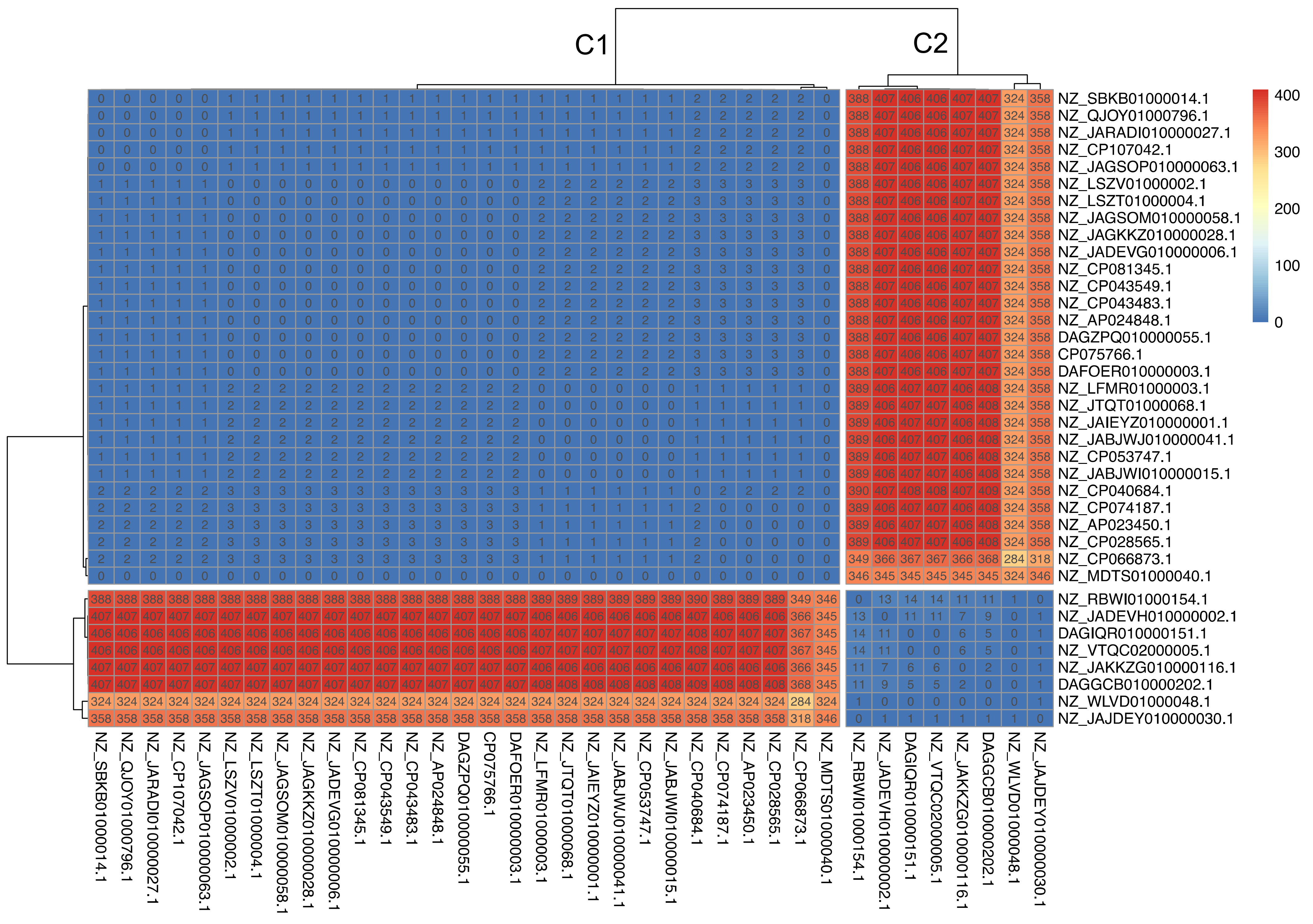
C1
C2
400
300
200
100
0
NZ_SBKB01000014.1
NZ_QJOY01000796.1
NZ_JARADI010000027.1
NZ_CP107042.1
NZ_JAGSOP010000063.1
NZ_LSZV01000002.1
NZ_LSZT01000004.1
NZ_JAGSOM010000058.1
NZ_JAGKKZ010000028.1
NZ_JADEVG010000006.1
NZ_CP081345.1
NZ_CP043549.1
NZ_CP043483.1
NZ_AP024848.1
DAGZPQ010000055.1
CP075766.1
DAFOER010000003.1
NZ_LFMR01000003.1
NZ_JTQT01000068.1
NZ_JAIEYZ010000001.1
NZ_JABJWJ010000041.1
NZ_CP053747.1
NZ_JABJWI010000015.1
NZ_CP040684.1
NZ_CP074187.1
NZ_AP023450.1
NZ_CP028565.1
NZ_CP066873.1
NZ_MDTS01000040.1
NZ_RBWI01000154.1
NZ_JADEVH010000002.1
DAGIQR010000151.1
NZ_VTQC02000005.1
NZ_JAKKZG010000116.1
DAGGCB010000202.1
NZ_WLVD01000048.1
NZ_JAJDEY010000030.1

Figure S4. Heatmap of integrase SNPs shows clusters for *intI1* (C1) and *intI3* (C2).

Numbers in cells indicate the number of SNPs between integrase pairs.

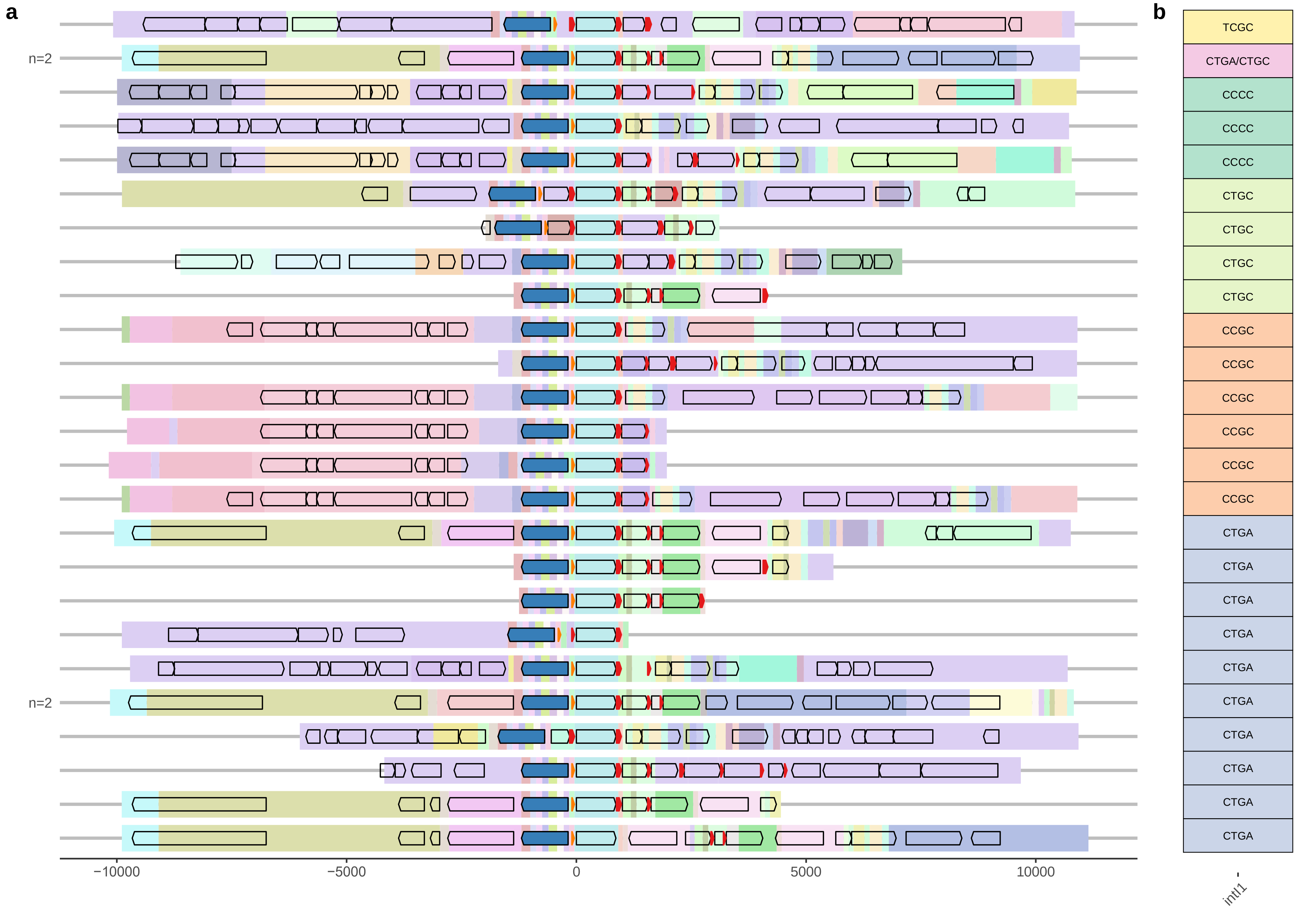

a
b
n=2
n=2
−10000
−5000
0
5000
10000
TCGC
CTGA/CTGC
CCCC
CCCC
CCCC
CTGC
CTGC
CTGC
CTGC
CCGC
CCGC
CCGC
CCGC
CCGC
CCGC
CTGA
CTGA
CTGA
CTGA
CTGA
CTGA
CTGA
CTGA
CTGA
CTGA
intI1

Figure S5. Full-length flanking sequences.

(a) 10,000bp *bla*$_{GES-5}$ flanking sequences containing *intI1* (blue arrow, flanking sequences subset from Figure 4) **(b)** grouped by *intI1* SNV profile.
